# Supplementary material for: Robust interface and reduced operation pressure enabled by co-rolling dry-process for stable all-solid-state batteries
Source: Nat Commun. 2025 May 6;16:4200. doi: 10.1038/s41467-025-59363-4 (PMC12055973; doi:10.1038/s41467-025-59363-4)
Supplement: Supplementary file 1 — Supplementary Information [file 41467_2025_59363_MOESM1_ESM.pdf]

Supplementary Information

Robust interface and reduced operation pressure enabled by co-rolling dry-process for stable all-solid-state batteries

Dong Ju Lee[1], Yuju Jeon[1], Jung-Pil Lee[2], Lanshuang Zhang[3], Ki Hwan Koh[1], Feng Li[1], Anthony U. Mu[1], Junlin Wu[3], Yu-Ting Chen[3], Seamus McNulty[1], Wei Tang[1], Marta Vicencio[1], Dapeng Xu[1], Jiyoung Kim[2], Zheng Chen[1,3,4*]

[1] Aiiso Yufeng Li Family Department of Chemical and Nano Engineering, University of California, San Diego, 9500 Gilman Drive, La Jolla, CA 92093, USA

[2] LG Energy Solution, Ltd. LG Science Park, Magokjungang 10-ro, Gangseo-gu, Seoul 07796, Republic of South Korea

[3] Program of Materials Science and Engineering, University of California, San Diego, 9500 Gilman Drive, La Jolla, CA 92093, USA

[4] Sustainable Power and Energy Center, University of California, San Diego, La Jolla, CA 92093, USA

[*] Corresponding author: zhc199@ucsd.edu

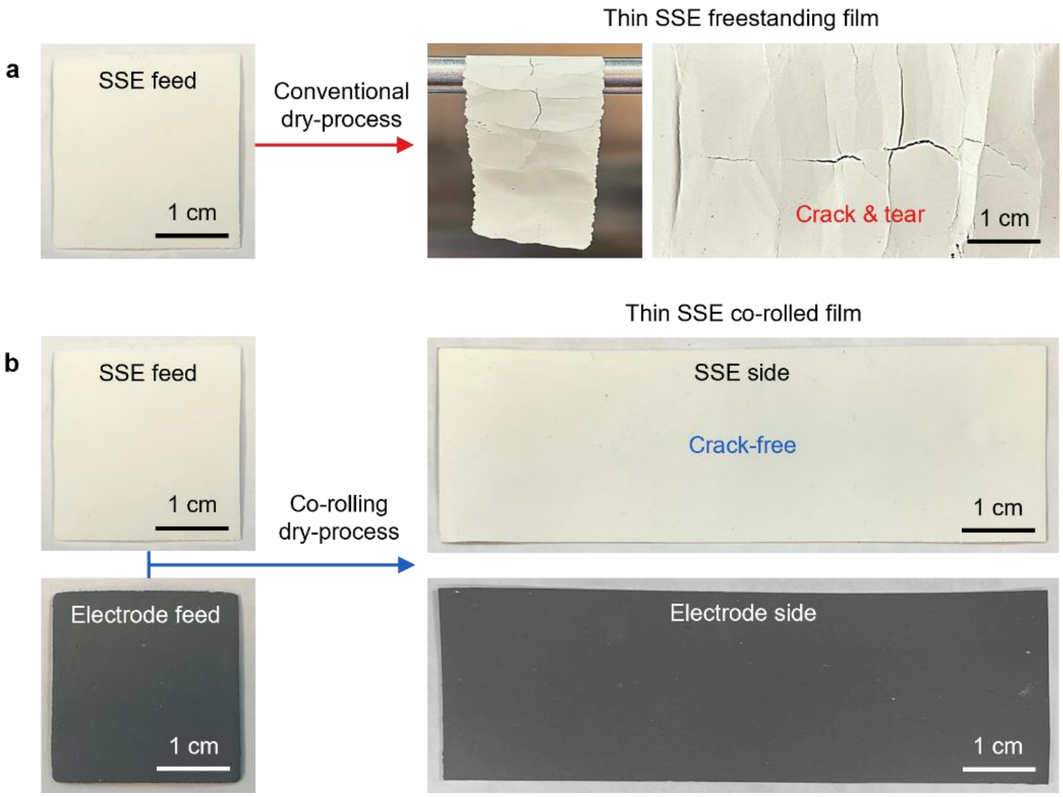

Supplementary Fig. 1 | Conventional dry-process and co-rolling dry-process. Photos of **(a)** a thin SSE freestanding film fabricated with conventional dry-process showing severe crack and tear and **(b)** a thin SSE co-rolled film fabricated co-rolling dry-process showing SSE and positive electrode sides with crack-free surface.

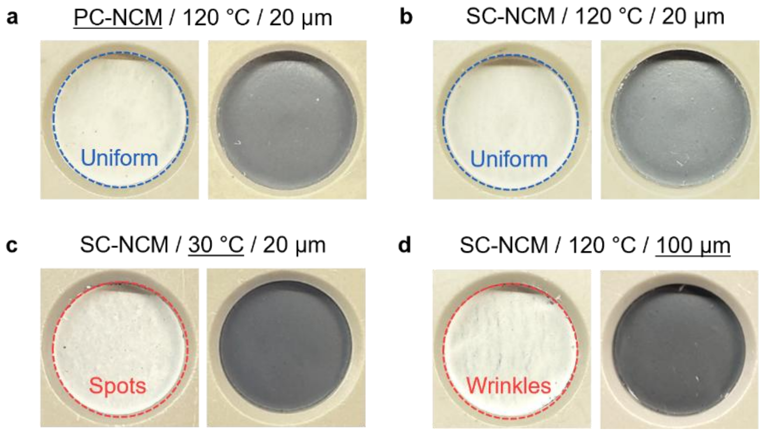

Supplementary Fig. 2 | Photos of SSE (bright yellow) and positive electrode (gray) sides of co-rolled film prepared with different fabrication parameters after press. (a) PC-NCM / 120 °C / 20 μm, **(b)** SC-NCM / 120 °C / 20 μm, **(c)** SC-NCM / 30 °C / 20 μm, and **(d)** SC-NCM / 120 °C / 100 μm. The slight difference in color is due to different angles and intensity of light.

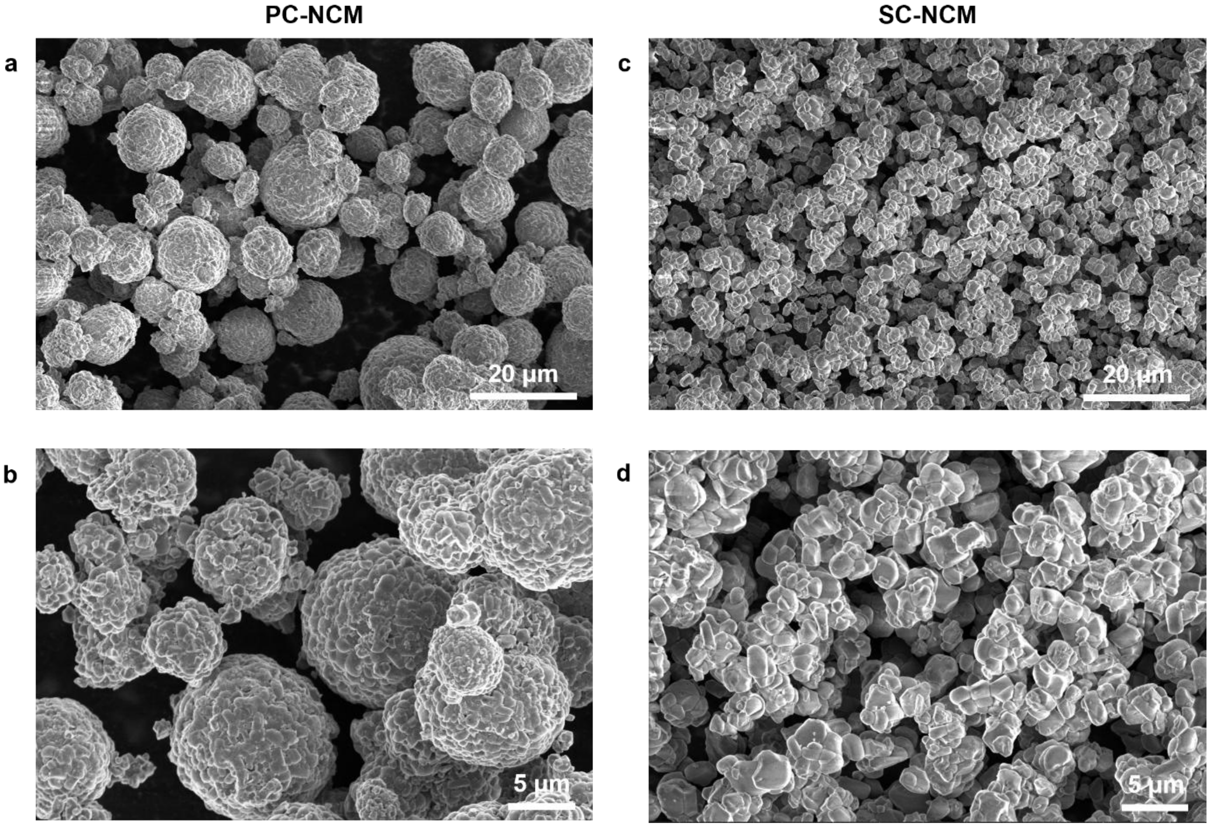

Supplementary Fig. 3 | Particle size of PC-NCM and SC-NCM. SEM images of **(a, b)** PC-NCM and **(c, d)** SC-NCM at low and high magnification, respectively.

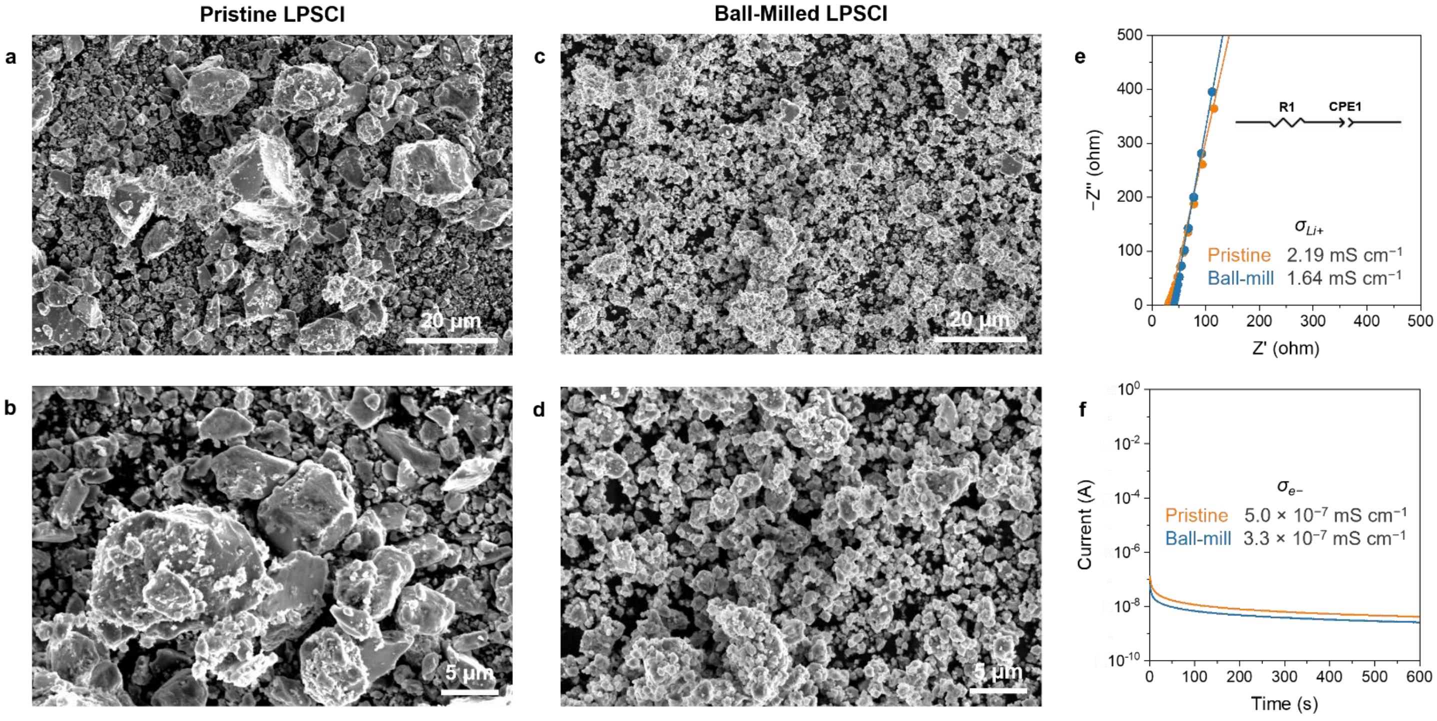

Supplementary Fig. 4 | Particle size and electrochemical properties of pristine and ball-milled LPSCl. SEM images of **(a, b)** pristine LPSCl and **(c, d)** ball-milled LPSCl at low and high magnification, respectively. **(e)** electrochemical impedance spectroscopy (EIS) of ionic conductivity measurement and **(f)** direct current polarization (DCP) of electronic conductivity measurement of pristine and ball-milled LPSCl. Both ionic and electronic conductivities were maintained after ball-milling. The tests were conducted at 23 ± 1 °C. Source data are provided as a Source Data file.

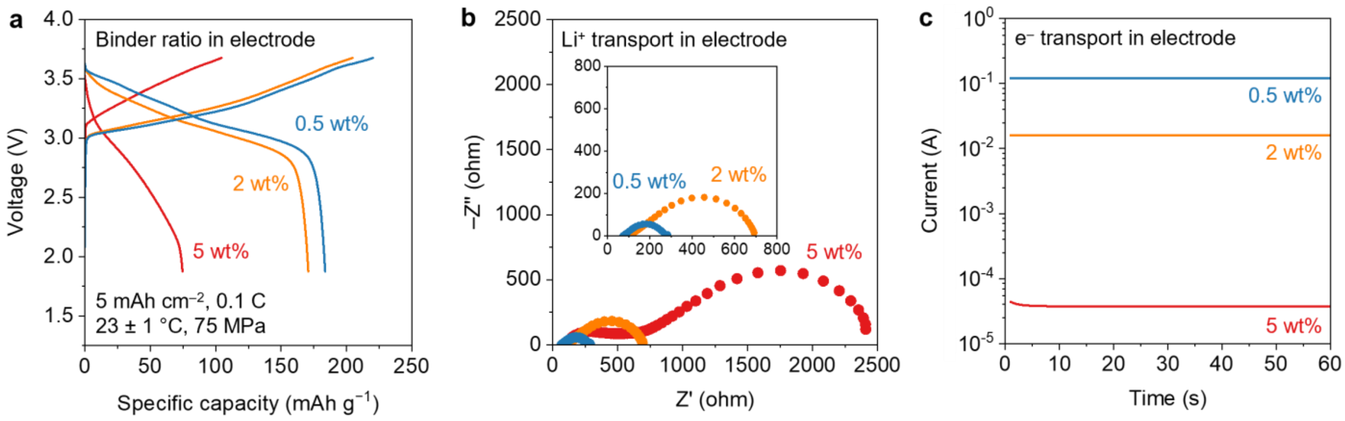

Supplementary Fig. 5 | Effect of PTFE binder ratio in positive electrode composite. (a) Voltage profiles of positive electrode films in LiIn|LPSCl|NCM configuration with different ratios of PTFE binder at 0.1 C (20 mA g^{-1}). **(b)** Li^+ transport and **(c)** e^- transport properties obtained from electron-blocking and electron-nonblocking cell configurations, respectively. The ionic conductivities were calculated to be 0.069, 0.024, 0.007 mS cm^{-1}, and electronic conductivities were calculated to be 34, 4.5, 0.011 mS cm^{-1} for PTFE ratios of 0.5, 2, 5 wt%, respectively. The weight ratio of CAM:SSE:VGCF was fixed to 80:17:3. Source data are provided as a Source Data file.

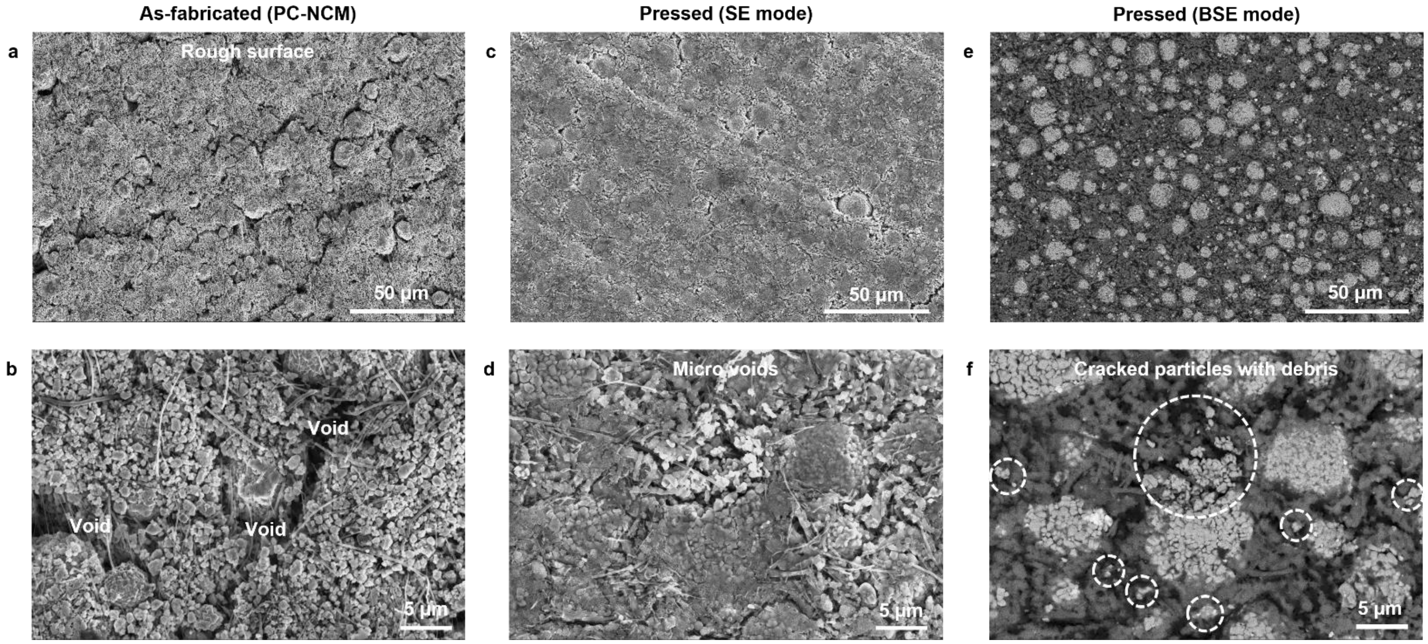

Supplementary Fig. 6 | Surface morphologies of co-rolled film with PC-NCM. SEM images of positive electrode side of co-rolled film with PC-NCM **(a, b)** as-fabricated, **(c, d)** pressed with secondary electron (SE) mode, and **(e, f)** pressed with back scattered electron (BSE) mode at low and high magnification, respectively.

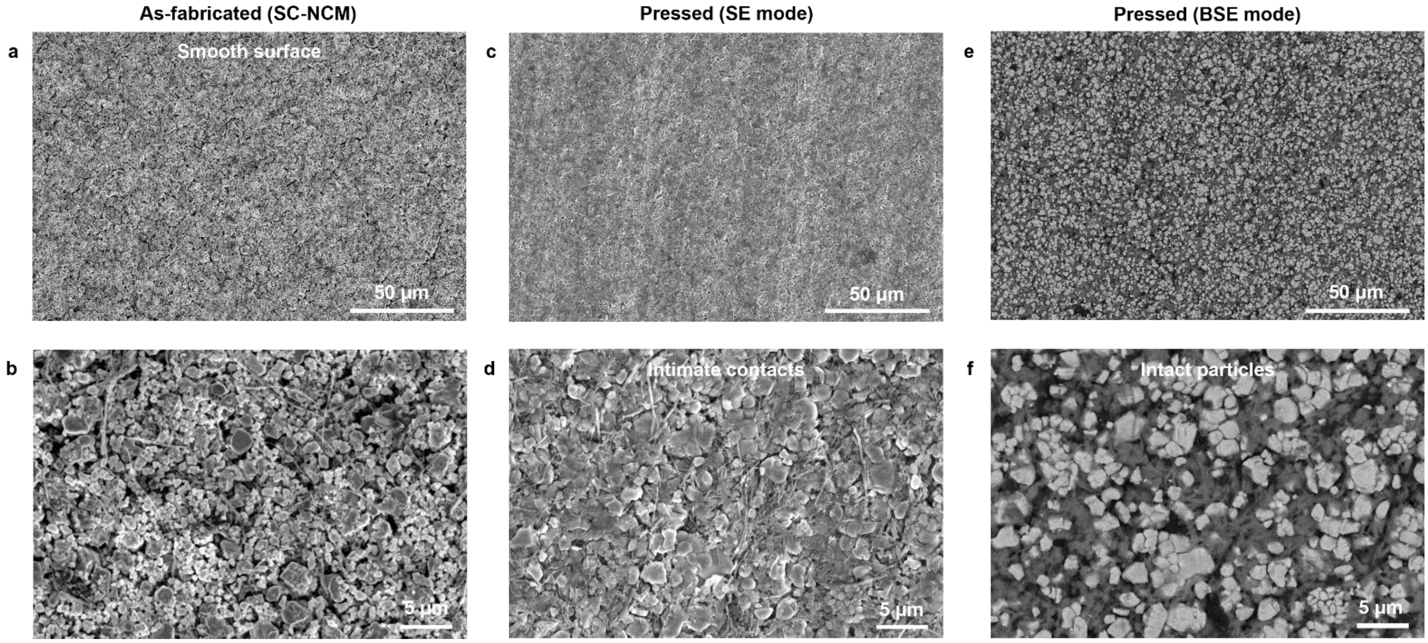

Supplementary Fig. 7 | Surface morphologies of co-rolled film with SC-NCM. SEM images of positive electrode side of co-rolled film with SC-NCM **(a, b)** as-fabricated, **(c, d)** pressed with SE mode, and **(e, f)** pressed with BSE mode at low and high magnification, respectively.

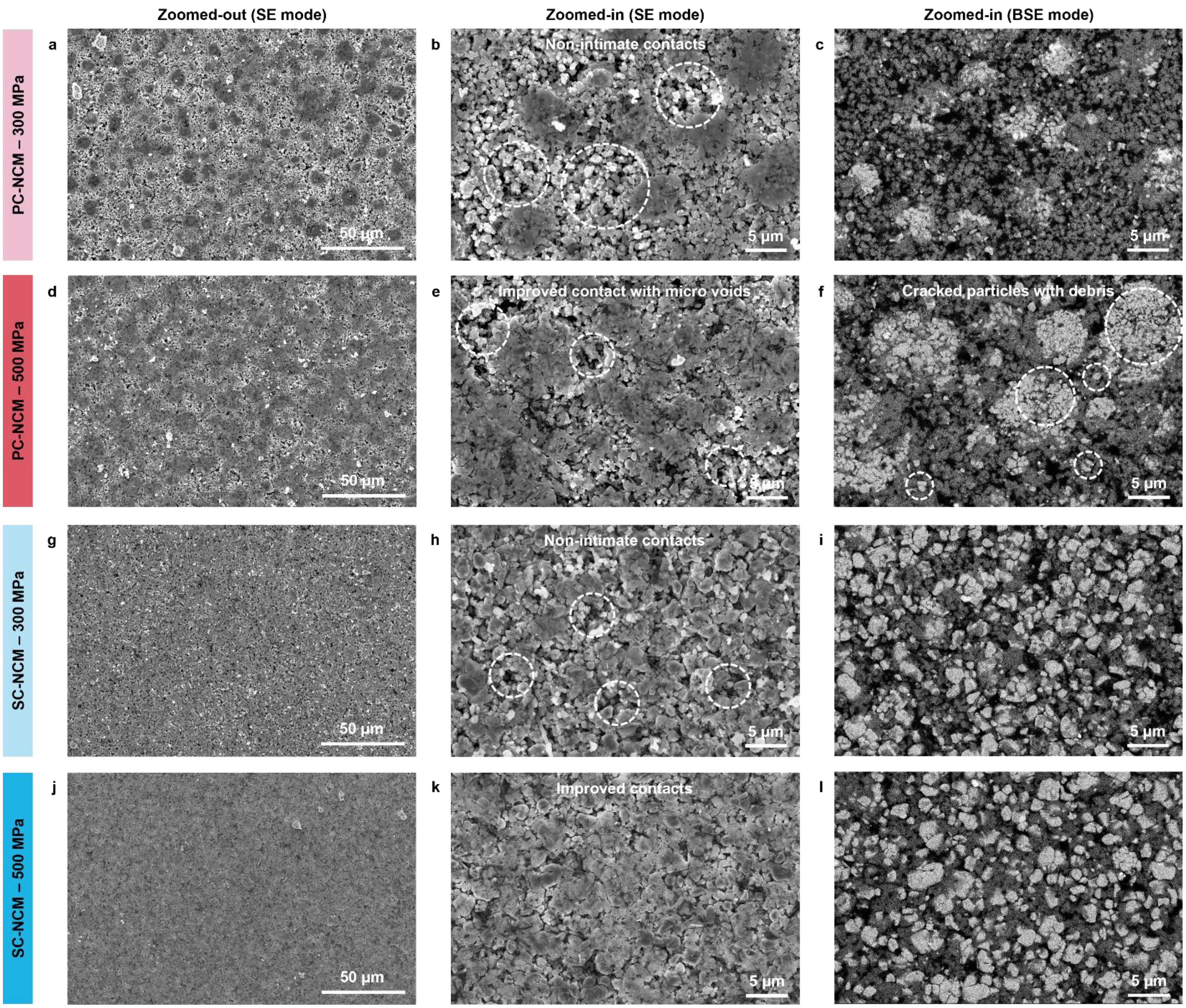

Supplementary Fig. 8 | Surface morphology of PC-NCM and SC-NCM at 300 and 500 MPa fabrication pressures. SEM images of powder positive electrode composite with PC-NCM fabricated at **(a-c)** 300 MPa and **(d-f)** 500 MPa and SC-NCM fabricated at **(g-i)** 300 MPa and **(j-l)** 500 MPa.

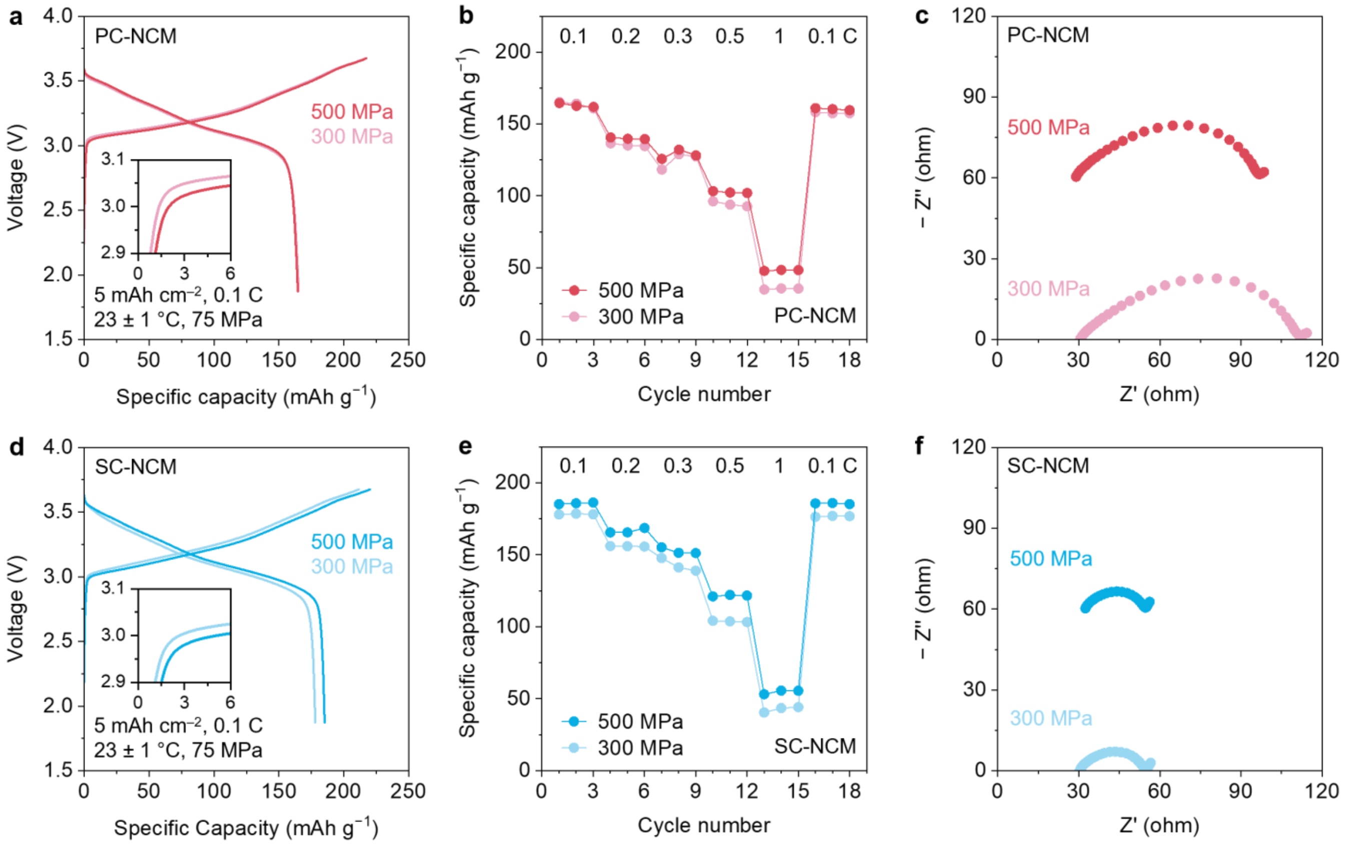

Supplementary Fig. 9 | Electrochemical properties of PC-NCM and SC-NCM at 300 and 500 MPa fabrication pressures. (a, d) Voltage profiles at 0.1 C (20 mA g^{-1}), **(b, e)** rate test at 0.1, 0.2, 0.3, 0.5, 1 C (20, 40, 60, 100, 200 mA g^{-1}, respectively), and **(c, f)** EIS of PC-NCM and SC-NCM in LiIn|LPSCl|NCM configuration, respectively, fabricated at 300 and 500 MPa. SC-NCM fabricated at 500 MPa showed the lowest polarization, highest rate capability, and lowest impedance. Source data are provided as a Source Data file.

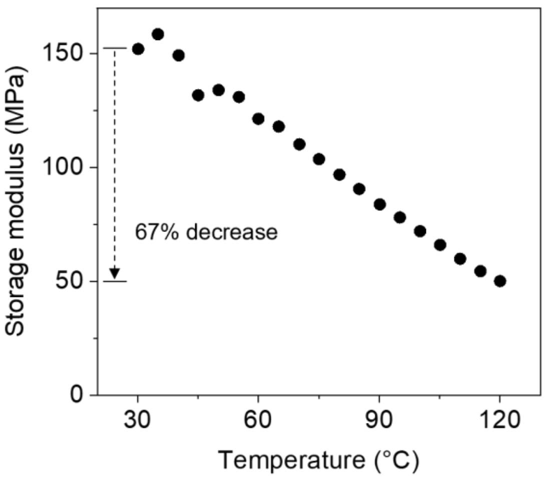

Supplementary Fig. 10 | Dynamic mechanical analysis (DMA) measurement of PTFE. 67% decrease in storage modulus is obtained from 30 to 120 °C. Source data are provided as a Source Data file.

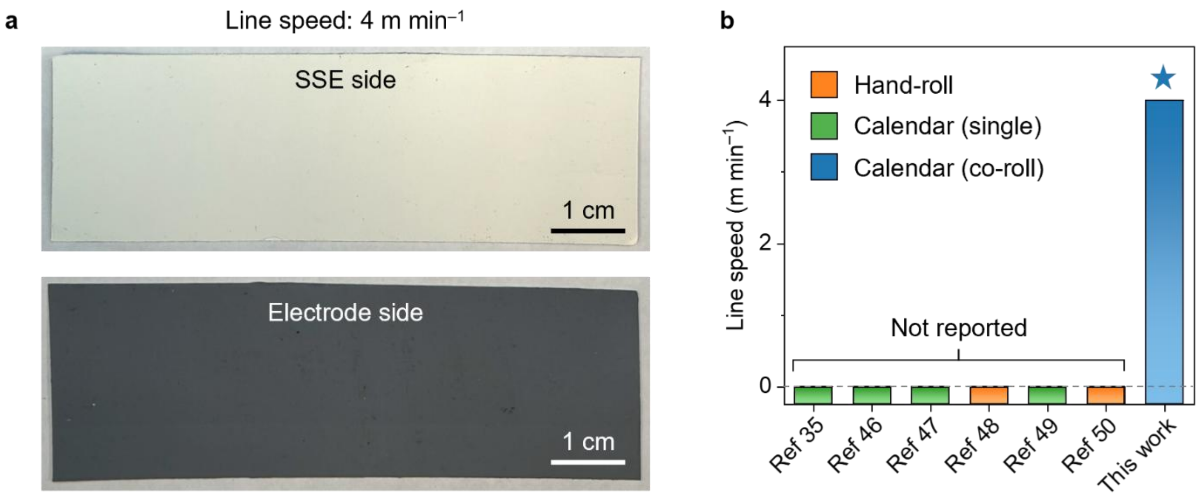

Supplementary Fig. 11 | Potential high-throughput capability of co-rolling dry-process. (a) Photos of co-rolled film fabricated at line speed of 4 m min^{-1}. **(b)** Comparison of line speed of thin dry-processed SSE layer with other published works. Note that this work is the first to consider the fabrication speed in dry-processed SSE films.

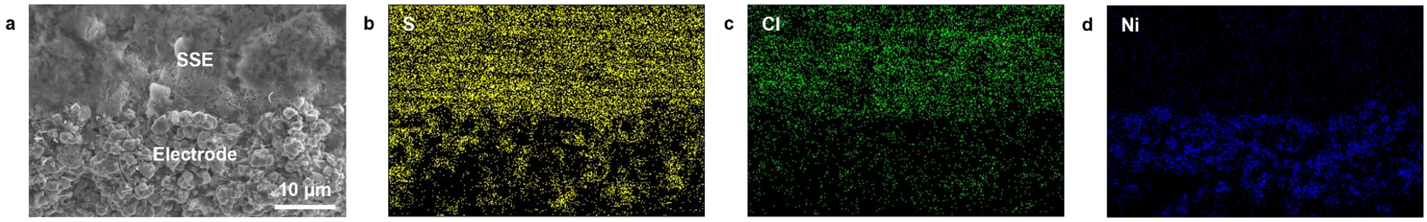

Supplementary Fig. 12 | Cross-sectional interphase analysis of co-rolled film. (a) SEM image and **(b-d)** EDS mapping of SSE-positive electrode interface of co-rolled film after press.

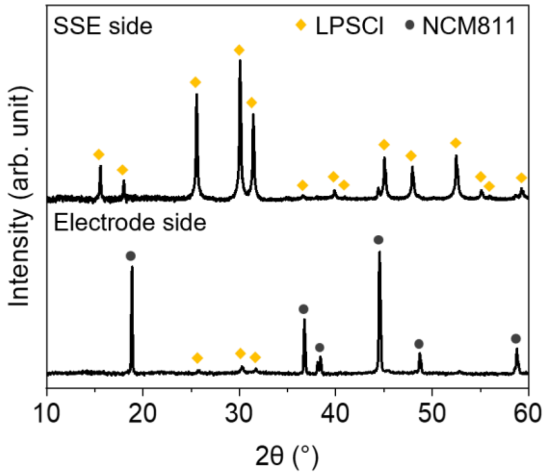

Supplementary Fig. 13 | XRD patterns of SSE side and positive electrode side of co-rolled film. Source data are provided as a Source Data file.

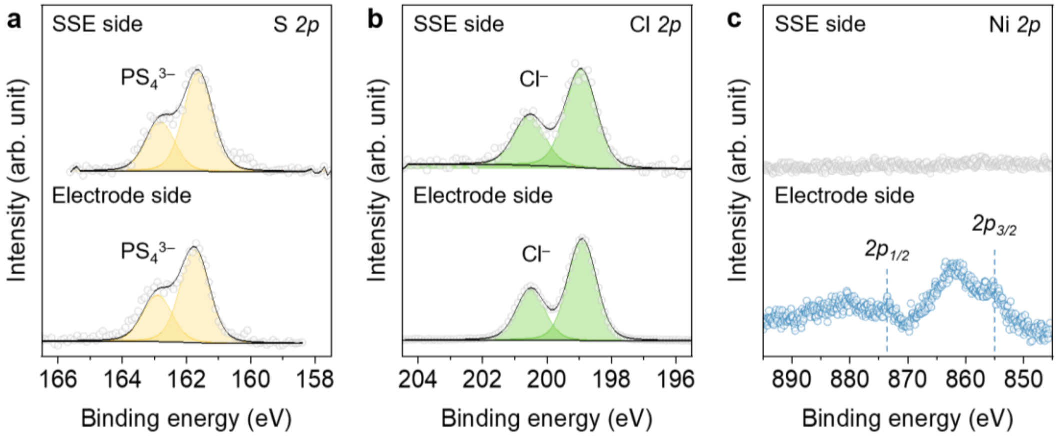

Supplementary Fig. 14 | XPS analysis of SSE and positive electrode sides of co-rolled film. (a) S *2p*, **(b)** Cl *2p*, **(c)** Ni *2p* spectra of SSE side and positive electrode side of co-rolled film. Source data are provided as a Source Data file.

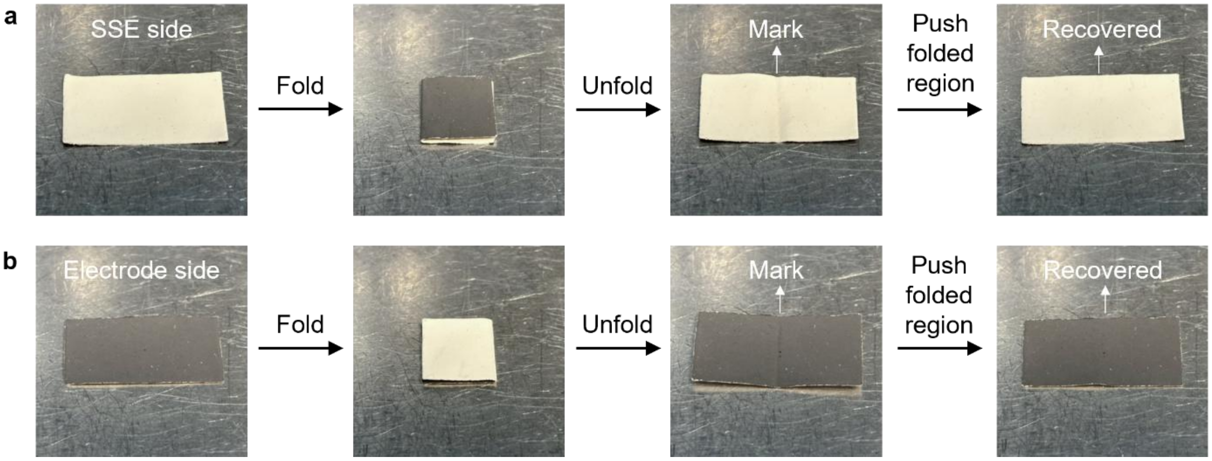

Supplementary Fig. 15 | Folding and unfolding test of co-rolled film. Photos of co-rolled films **(a)** folded inward SSE side and **(b)** folded inward positive electrode side.

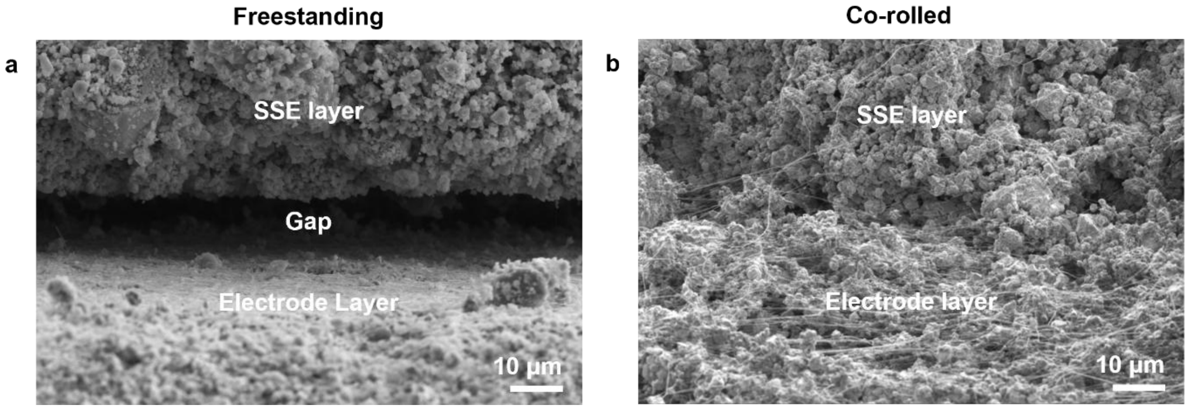

Supplementary Fig. 16 | Typical interface structure of freestanding co-rolled films. Side view SEM images of **(a)** laminated freestanding films and **(b)** co-rolled film after tearing.

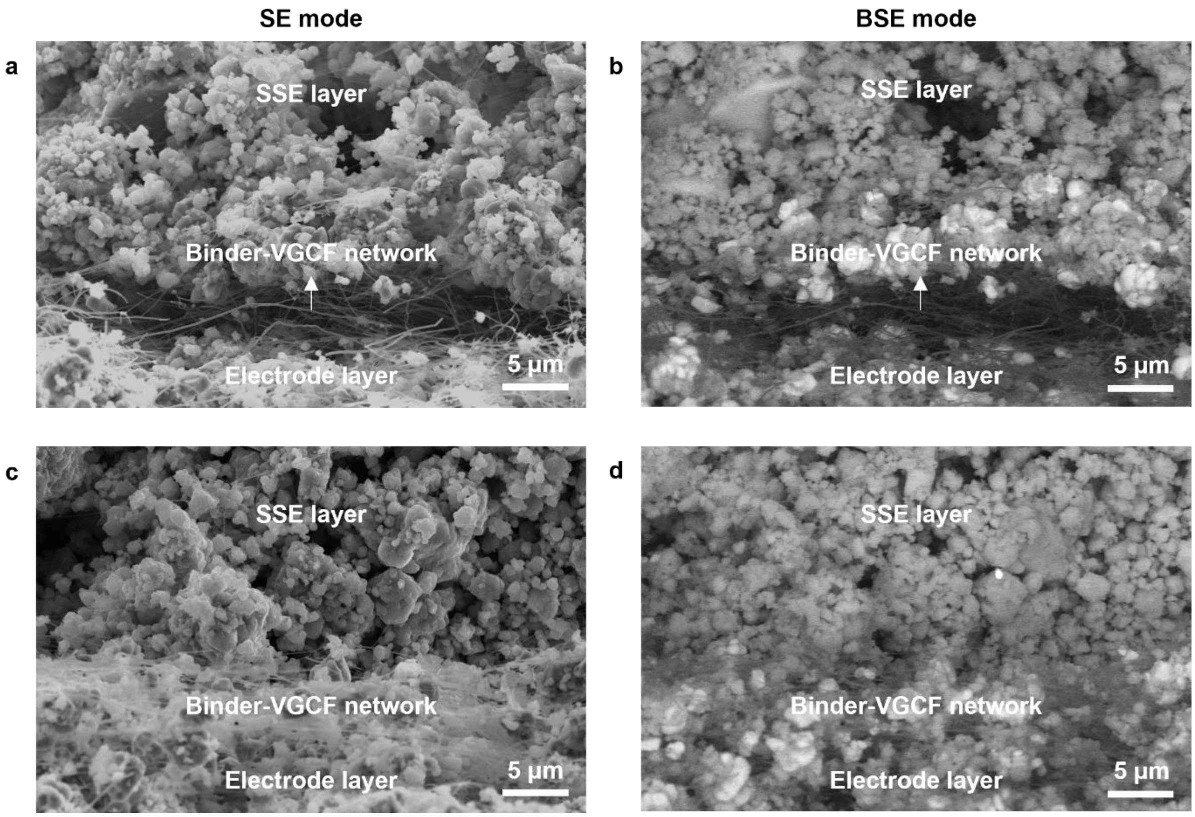

Supplementary Fig. 17 | Fibrillated network of binder-VGCF at the interface. Full size SEM images of co-rolled film in Fig. 3e with **(a)** SE mode and **(b)** BSE mode. SEM images of a typical attached region with **(c)** SE mode and **(d)** BSE mode.

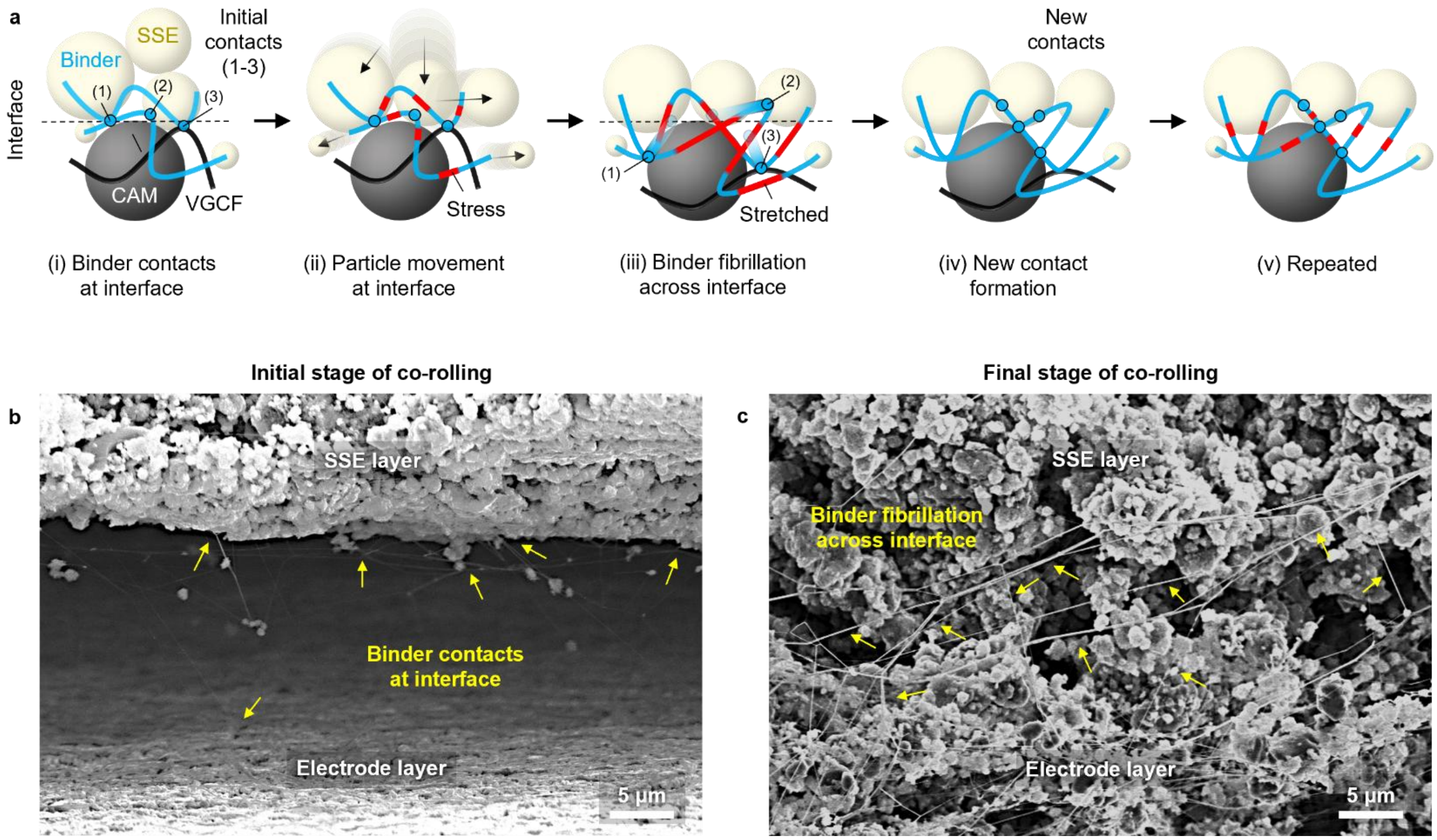

Supplementary Fig. 18 | Binder fibrillation across the interface. **(a)** A proposed mechanism of interfacial fibrillation during co-rolling process. (i) Several different contacts can be formed at SSE-positive electrode interface (dashed line): (1) Binder in SSE layer-to-binder in positive electrode layer, (2) SSE particle in SSE layer-to-binder in positive electrode layer, and (3) binder in SSE layer-to-VGCF in positive electrode layer. (ii) Shearing induces movement of materials at the interface as well as stress on the binder. (iii) Due to the movement, binders are stretched and fibrillated across the interface. As a result, the initial contacts (1-3) have moved across the interface. (iv) New contacts have formed from the interfacial fibrillation of binder. (v) This process is repeated with every step of thickness reduction. SEM images of SSE-positive electrode interface during **(b)** initial stage of co-rolling showing binder contacts at the interface and **(c)** final stage of co-rolling showing binder fibrillation across the interface.

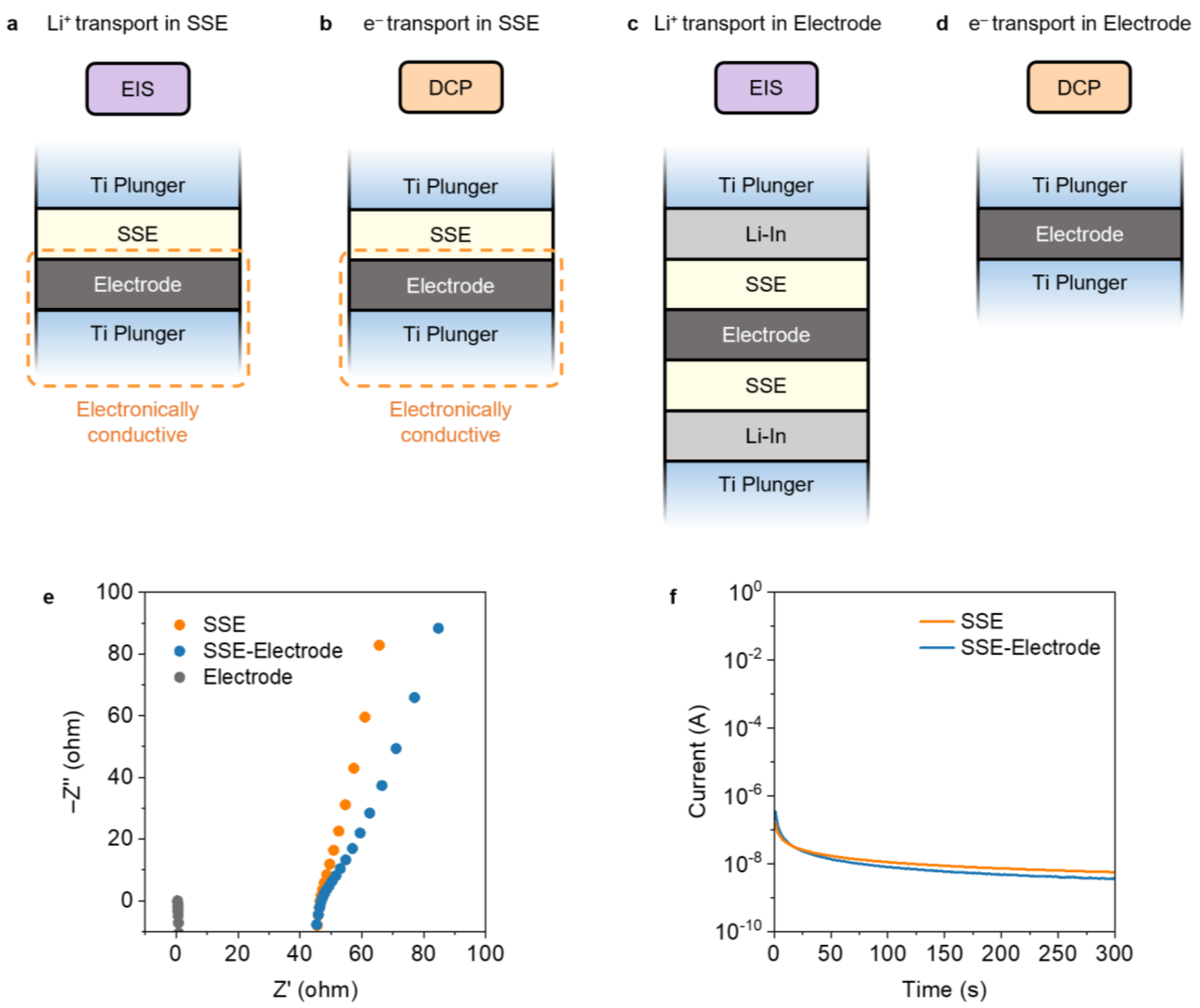

Supplementary Fig. 19 | Electrochemical characterizations of co-rolled film. Cell configurations and electrochemical methods used for the characterization of **(a)** Li^+ transport in SSE, **(b)** e^- transport in SSE, **(c)** Li^+ transport in positive electrode, and **(d)** e^- transport in positive electrode. Comparison of measurement results on **(e)** EIS and **(f)** DCP between SSE and SSE|electrode configurations. Li^+ and e^- transports in SSE were conducted with SSE|electrode structure due to the intrinsically integrated structure of co-rolled film. The high e^- conductivity of electrode layer (> 30 mS cm^{-1}) showed negligible impacts on the bulk impedance of EIS for Li^+ transport in SSE and the current measured of DCP for e^- transport in SSE. For Li^+ transport in positive electrode, powder SSE was used for freestanding films, and partial power SSE and SSE layer of co-rolled film were used for co-rolled film. The overall thickness of SSE layers was fixed. For e^- transport in positive electrode, SSE layer of co-rolled film was carefully separated from positive electrode layer by peeling-off with tapes. The tests were conducted at 23 ± 1 °C. Source data are provided as a Source Data file.

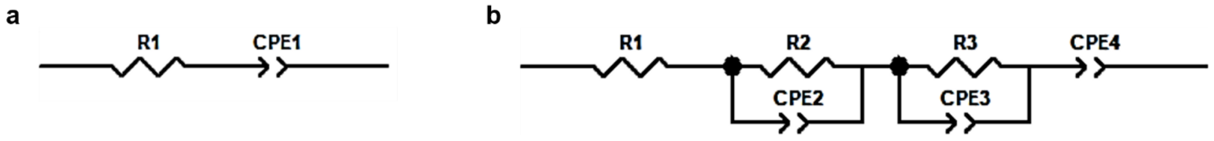

Supplementary Fig. 20 | Equivalent circuits used to analyze EIS results. (a) Li^+ transport in SSE in Fig. 4c and **(b)** Li^+ transport in positive electrode in Fig. 4e.

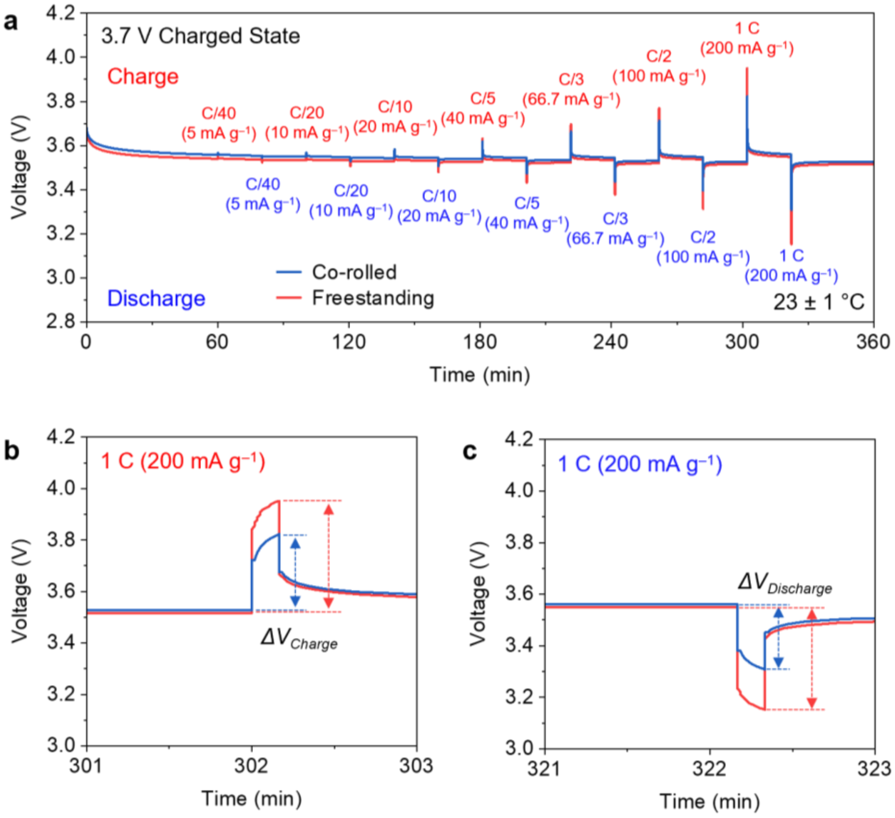

Supplementary Fig. 21 | Internal resistance test. (a) Voltage curves of internal resistance test of co-rolled and freestanding films in Si|LPSCl|NCM configuration with respect to time in Fig. 4g. Zoomed-in voltage responses of **(b)** 1C (200 mA g^{-1}) charge and **(c)** 1C (200 mA g^{-1}) discharge. The tests were conducted at 23 ± 1 °C. Source data are provided as a Source Data file.

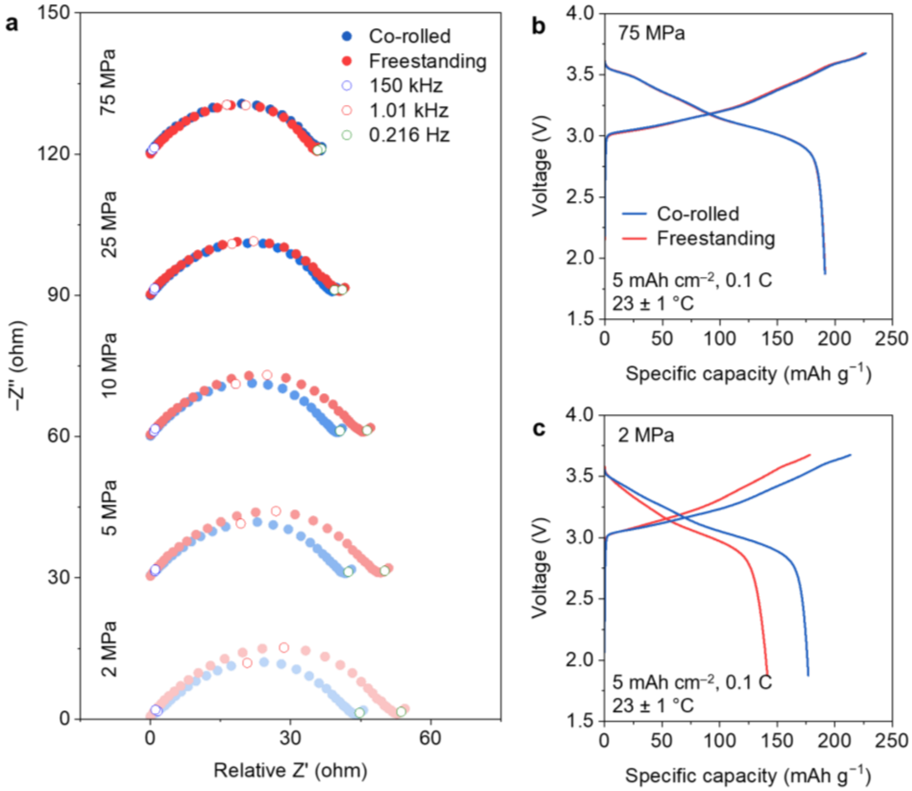

Supplementary Fig. 22 | Stack pressure test. (a) EIS measurements obtained at different stack pressures in Fig. 4i. Voltages profiles of co-rolled and freestanding films in LiIn|LPSCl|NCM configuration cycled at 0.1 C (20 mA g^{-1}) with stack pressures of **(b)** 75 MPa and **(c)** 2 MPa. Source data are provided as a Source Data file.

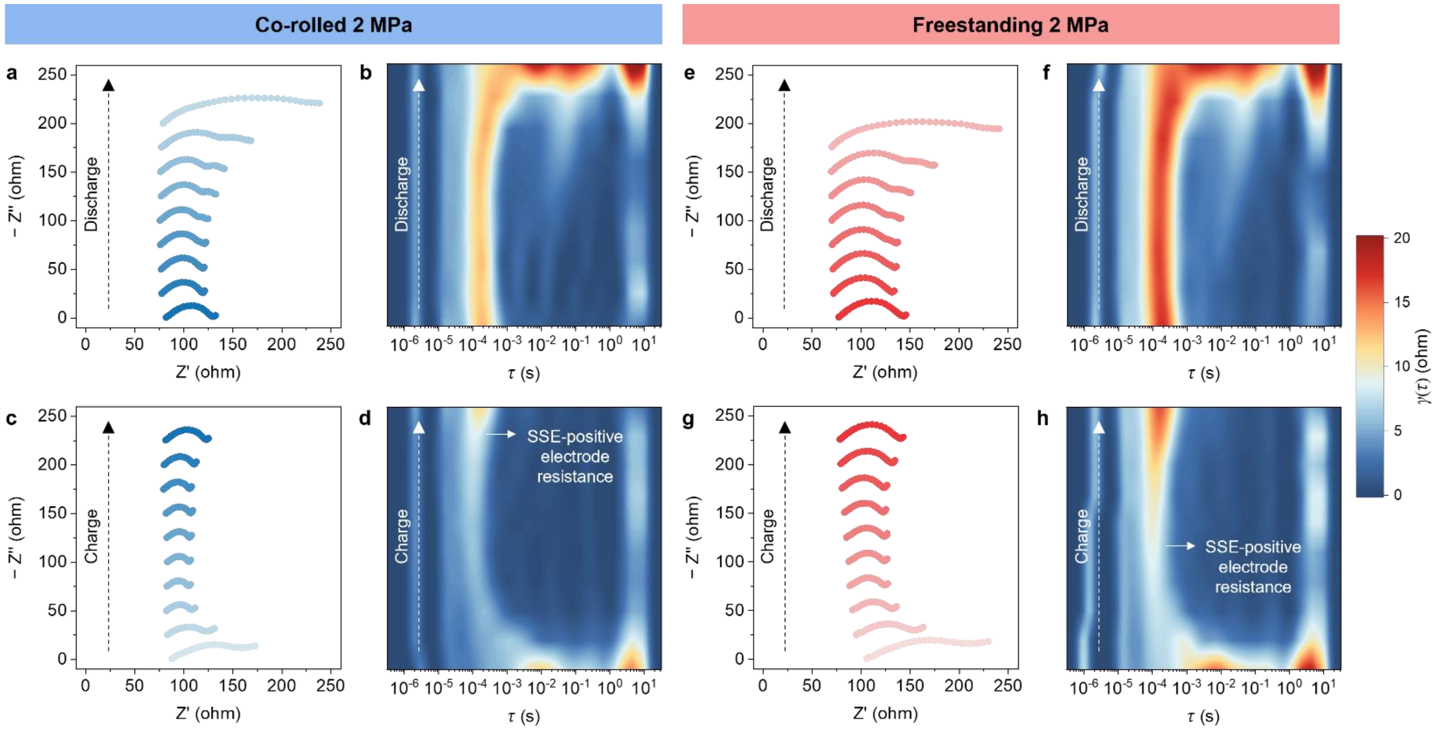

Supplementary Fig. 23 | In situ observation of SSE-positive electrode resistance evolution of co-rolled films and freestanding films at 2 MPa. EIS measurement and DRT analysis, respectively, of **(a, b)** discharge process and **(c, d)** charge process of co-rolled film. EIS measurement and DRT analysis, respectively, of **(e, f)** discharge process and **(g, h)** charge process of freestanding films. Half-cells using LiIn|LPSCl|NCM configuration were cycled at 0.1 C (20 mA g^{-1}) at 23 °C. Source data are provided as a Source Data file.

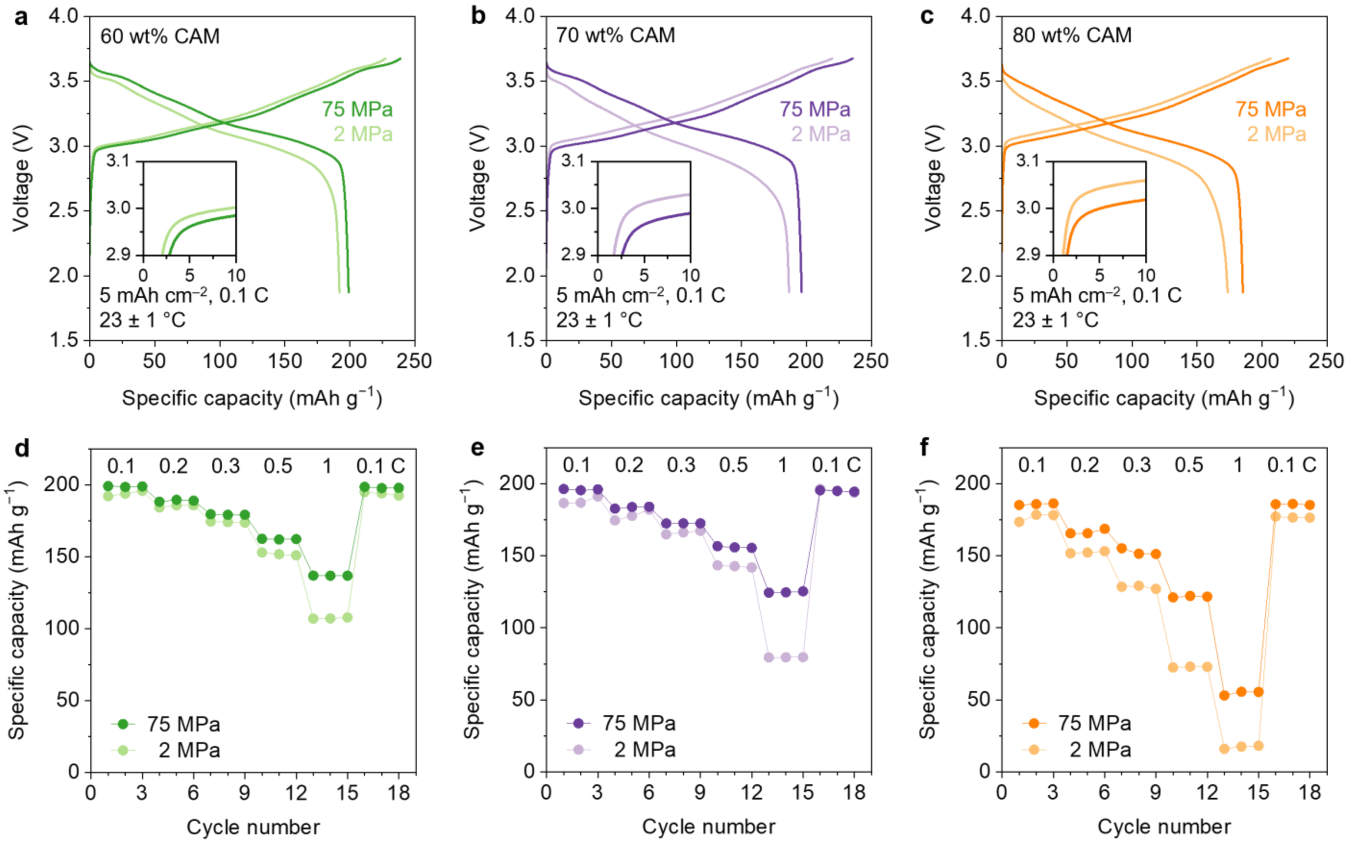

Supplementary Fig. 24 | Electrochemical performance of different CAM ratios at 75 and 2 MPa. (a-c) Voltage profiles at 0.1 C (20 mA g^{-1}) and **(d-f)** rate test at 0.1, 0.2, 0.3, 0.5, 1 C (20, 40, 60, 100, 200 mA g^{-1}, respectively) of different CAM ratios of 60, 70, and 80 wt%, respectively, in LiIn|LPSCl|NCM configuration. Source data are provided as a Source Data file.

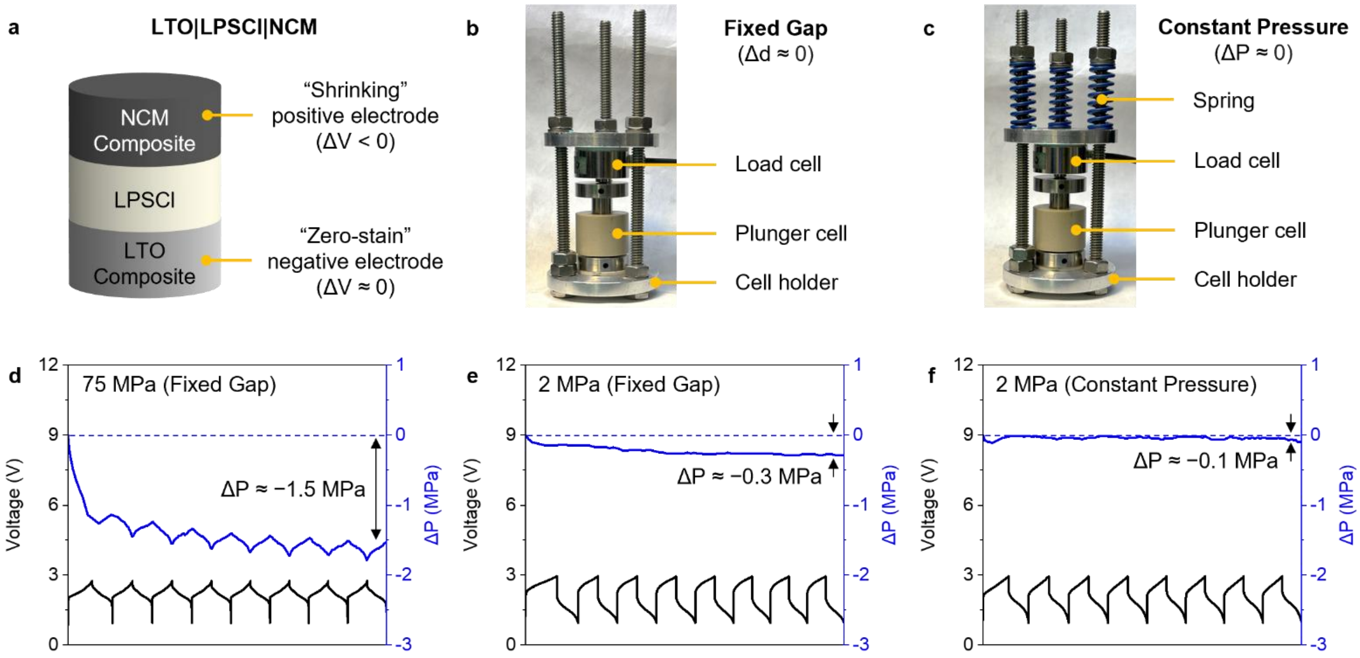

Supplementary Fig. 25 | Cell design and pressurization methods used to evaluate long-term cycling stability of SSE-positive electrode structures. **(a)** A schematic of LTO|LPSCl|NCM configuration with their volume change during charging. Photos of **(b)** fixed gap setup and **(c)** constant pressure setup. Monitored pressure changes during cell operation of **(d)** 75 MPa – fixed gap, **(e)** 2 MPa – fixed gap, and **(f)** 2 MPa – constant pressure setups. For long-term cycling stability test at different stack pressures, the fixed gap setup was used for 75 MPa, and the constant pressure setup was used for 2 MPa. Note that the constant pressure setup could not be used for 75 MPa due to the lack of suitable springs to sustain such high pressure. The cells were cycled at 0.1 C (20 mA g^{-1}) at 23 °C. Source data are provided as a Source Data file.

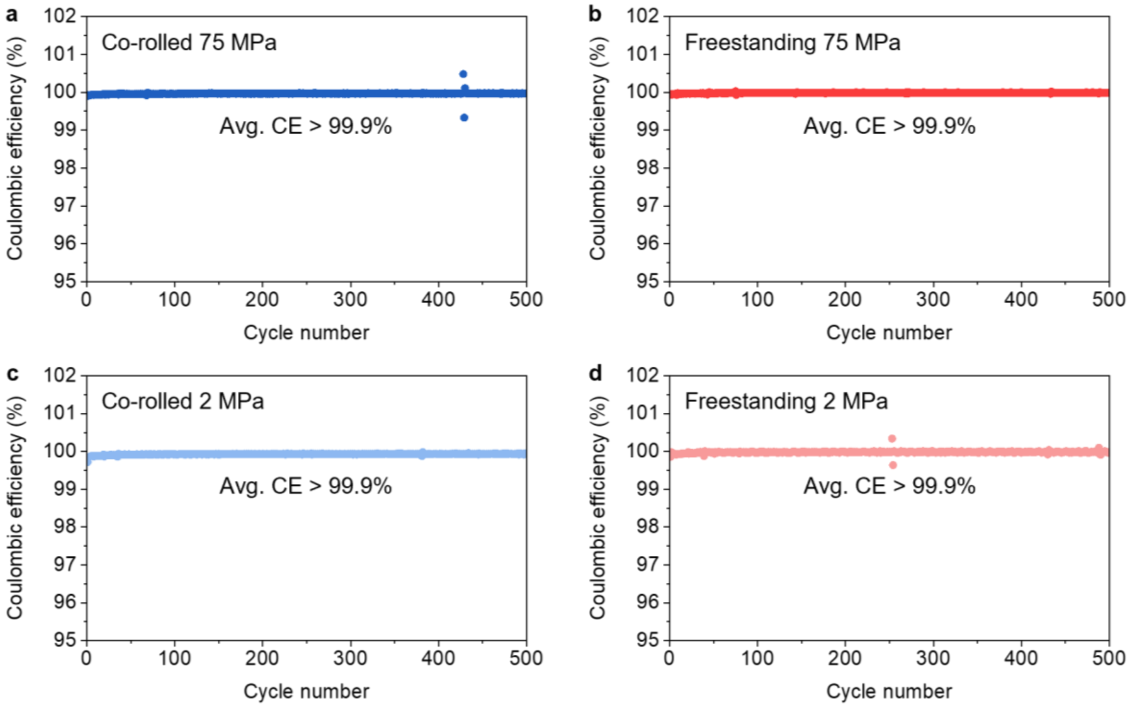

Supplementary Fig. 26 | Coulombic efficiencies of long-term cycling test. (a) Co-rolled film at 75 MPa, **(b)** freestanding films at 75 MPa, **(c)** co-rolled film at 2 MPa, and **(d)** freestanding films at 2 MPa. The average Coulombic efficiencies were all over 99.9% for 500 cycles. Source data are provided as a Source Data file.

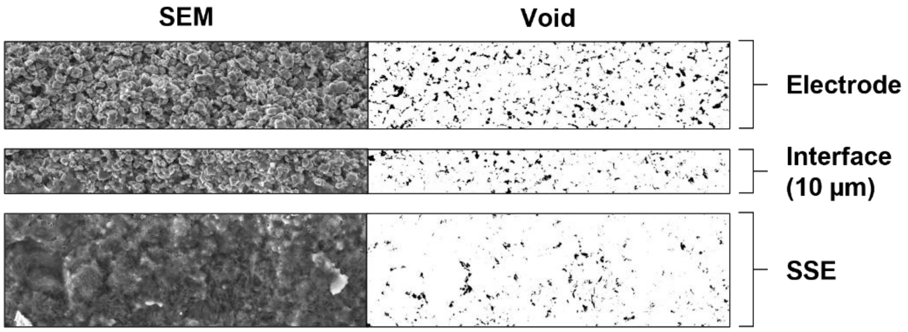

Supplementary Fig. 27 | An example of region assignment of positive electrode, interface, and SSE layers used for void analysis. The length of interface layer was fixed to 10 μm from the SSE layer.

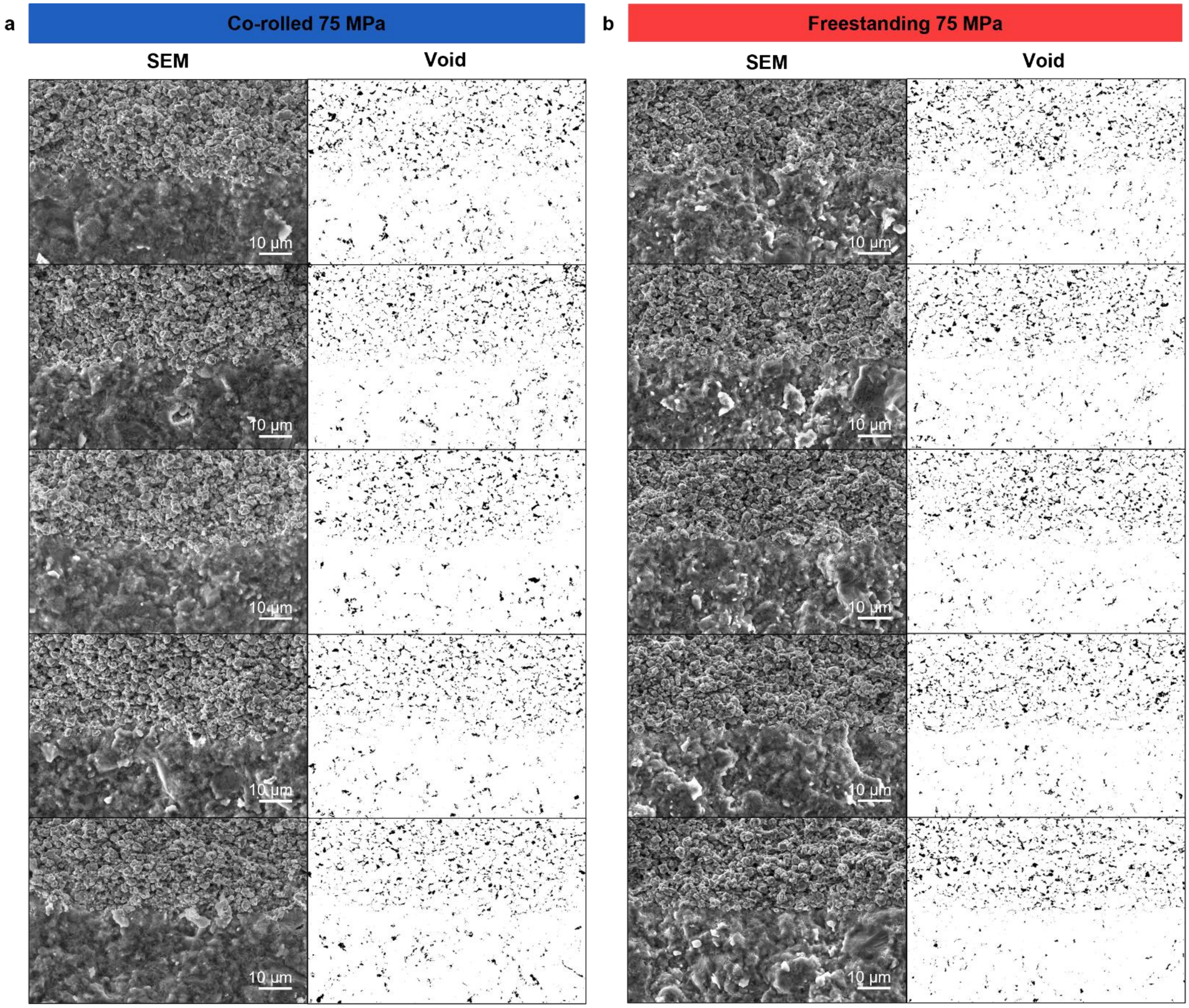

Supplementary Fig. 28 | Void analysis at 75 MPa. Cross-sectional SEM images and void segmentation of **(a)** co-rolled film and **(b)** freestanding films after cycling at stack pressure of 75 MPa. SEM images were taken at different regions of the same sample. Both co-rolled and freestanding films showed comparable void fraction both in positive electrode (top) and SSE (bottom) layers as well as intimate contact at the interface (middle) without obvious voids or cracks.

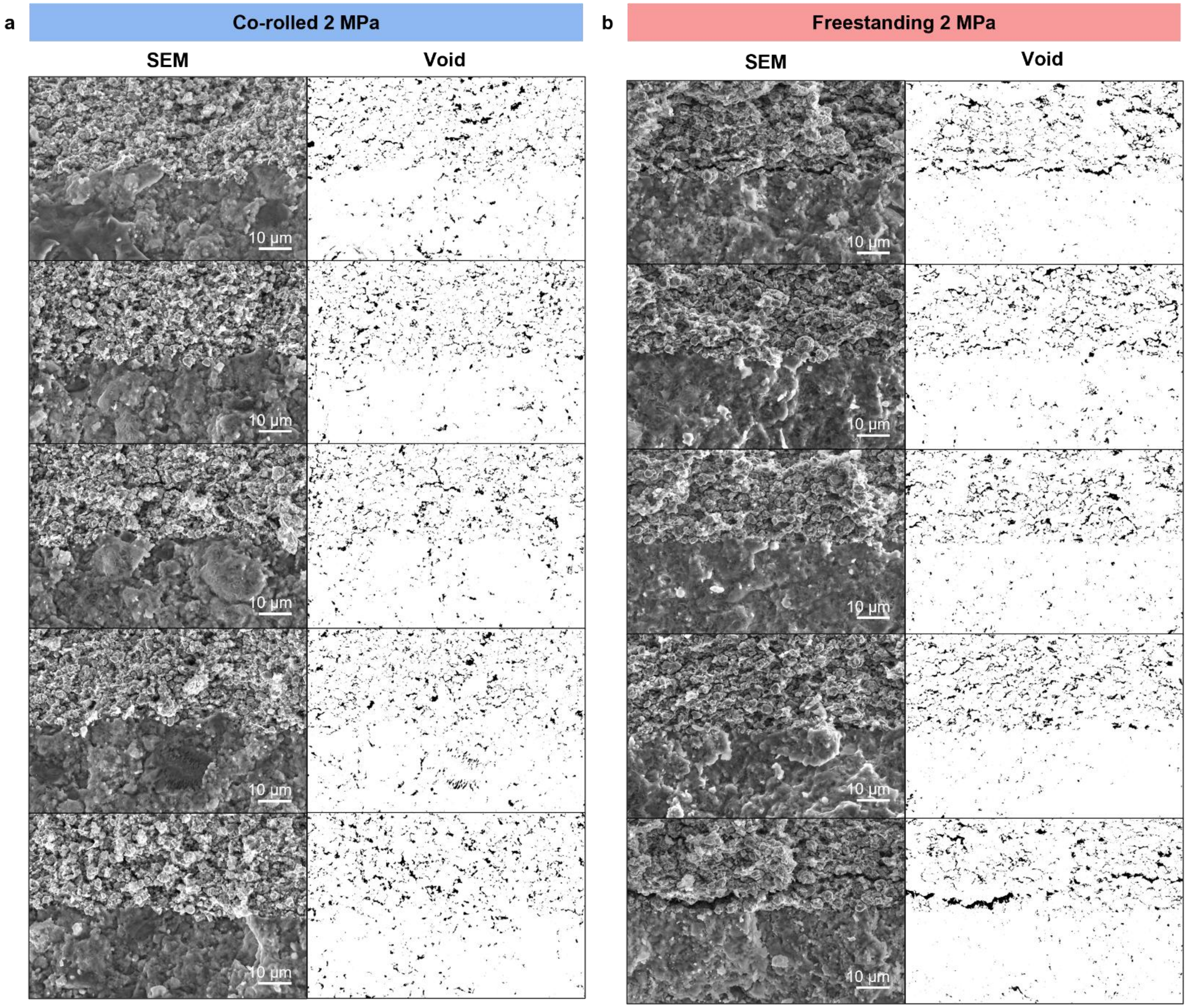

Supplementary Fig. 29 | Void analysis at 2 MPa. Cross-sectional SEM images and void segmentation of **(a)** co-rolled film and **(b)** freestanding films after cycling at stack pressure of 2 MPa. SEM images were taken at different regions of the same sample. While co-rolled film showed maintained contact at the interface (middle), freestanding films showed severe interfacial voids and cracks.

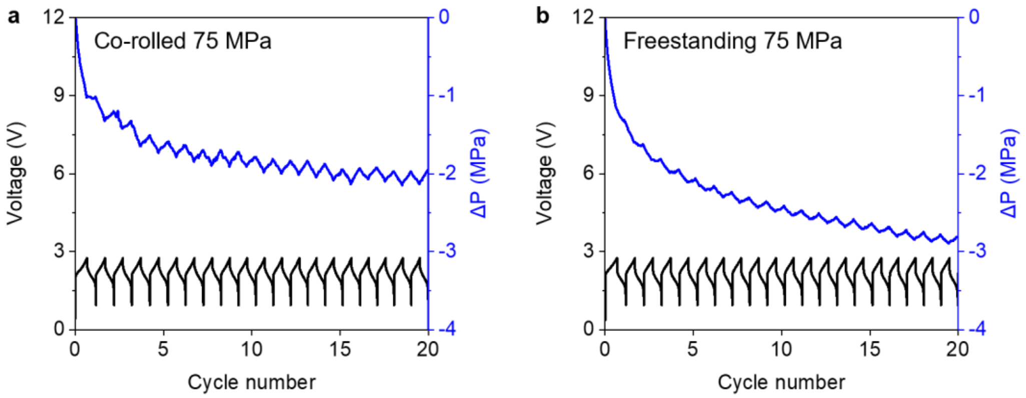

Supplementary Fig. 30 | Pressure changes of co-rolled film and freestanding films. Monitored pressure change of **(a)** co-rolled film and **(b)** freestanding films in LTO|LPSCl|NCM configuration at 75 MPa for 20 cycles. The cell with co-rolled film showed less pressure decrease than that with freestanding films (approximately −2 MPa vs. −3 MPa). The cells were cycled at 0.2 C (40 mA g^{-1}) at 23 °C. Source data are provided as a Source Data file.

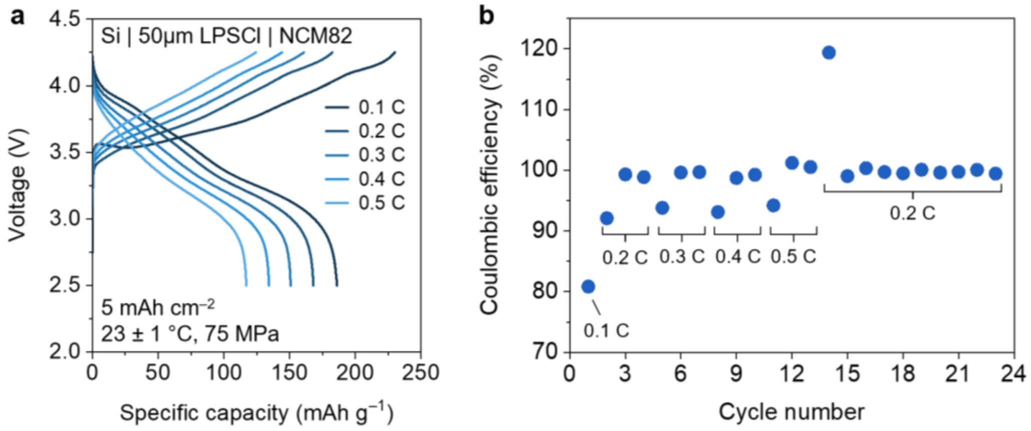

Supplementary Fig. 31 | Co-rolled film full-cell with Si. (a) Voltage profile and **(b)** Coulombic efficiency of data shown in Fig. 6b. The test was carried out at 0.1, 0.2, 0.3, 0.4, 0.5 C (20, 40, 60, 80, 100 mA g^{-1}, respectively). Source data are provided as a Source Data file.

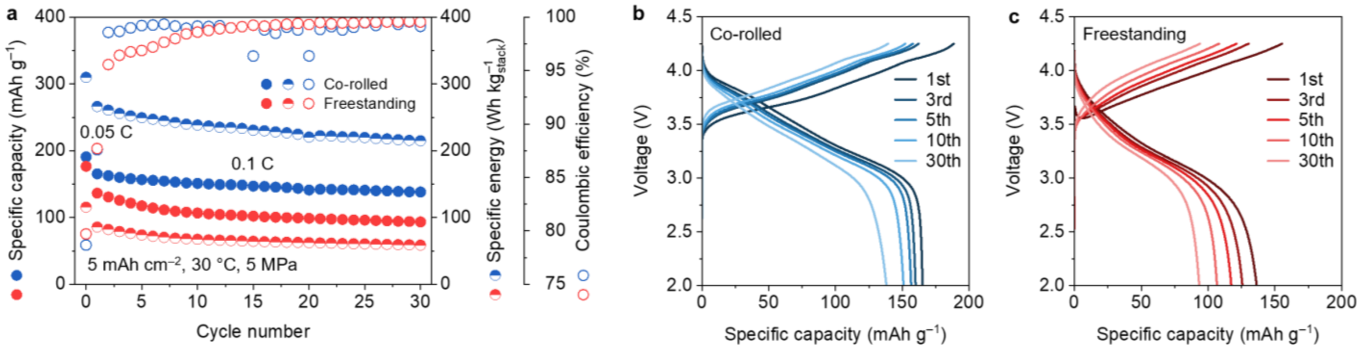

Supplementary Fig. 32 | Pouch cells of co-rolled film and freestanding films. (a) Comparison of cycling performance of pouch cells assembled with co-rolled film and freestanding films in Si|LPSCl|NCM configuration at 0.1C (20 mA g^{-1}) with activation at 0.05 C (10 mA g^{-1}). Voltage profiles of **(b)** co-rolled film and **(c)** freestanding films. Source data are provided as a Source Data file.

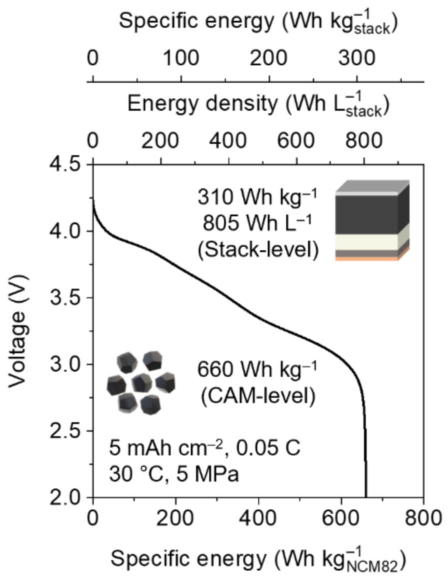

Supplementary Fig. 33 | Specific energy and energy density measurement of an ASSB pouch cell. The cell assembled with co-rolled film and Si was cycled at 0.05 C (10 mA g^{-1}). The stack-level (including positive electrode, SSE, negative electrode, and Al and Cu current collectors) specific energy and energy density are calculated to be 310 Wh kg^{-1} and 805Wh L^{-1}, respectively. Source data are provided as a Source Data file.

Supplementary Table 1 | Comparison of published works on dry-processed SSE layer.

		Ref 35	Ref 46	Ref 47	Ref 48	Ref 49	Ref 50	**This work**
**SSE**	**Material**	Li_6PS_5Cl	Li_6PS_5Cl + Li_3InCl_6	$Li_{5.4}PS_{4.4}Cl_{1.6}$	Li_6PS_5Cl	$Li_{10}GeP_2S_{12}$	Li_6PS_5Cl	Li_6PS_5Cl
	**Binder**	PTFE	PTFE	PTFE	PTFE	PTFE-Nylon mesh	PTFE	PTFE
	**Thickness (μm)**	150	40	30	48	100	42	50
	**SSE:Binder ratio**	40:00.2	99.5:0.5	99.8:0.2	100:0.4	100:1	99.8:0.2	100:0.1
	**Areal Mass (mg cm^{-2})**	24.6	8.4	4.92	7.872	20.4	6.56	8.2
**Positive electrode**	**Material**	NCM 9055	LCO	NCM 523	Thiuram sulfide	NCM 523	NCM 622	NCM 82
	**SSE**	Li_6PS_5Cl	Li_3InCl_6	$Li_{5.4}PS_{4.4}Cl_{1.6}$	Li_6PS_5Cl	$Li_{10}GeP_2S_{12}$	Li_6PS_5Cl	Li_6PS_5Cl
	**Binder**	PTFE	PTFE	PTFE	PTFE	-	PTFE	PTFE
	**Thickness (μm)**	86	86.5	66.1	88.0	34.4	28.2	120
	**AM:SSE:C:Binder ratio**	85:13:2:0.3	85:15:0:0.5	70:30:2:1	33.3:16.7:50: 0.8	65:25:10:0	60:40:3:0.5	80:17:3:0.5
	**Areal capacity (mAh cm^{-2})**	2.9	3.00	1.86	1.20	0.96	0.64	5
	**Areal mass (mg cm^{-2})**	17.1	25.3	17.1	6.1	9.2	6.9	31.4
**Negative electrode**	**Material**	Graphite	Graphite	Al_2O_3@Li	LiIn	GPE@Li	LiIn	Si
	**SSE**	Li_6PS_5Cl	Li_6PS_5Cl	-	-	-	-	-
	**Binder**	PTFE	PTFE	-	-	-	-	PVDF
	**Thickness (μm)**	83.2	107.9	80	277	100	100	15
	**AM:SSE:C:Binder ratio**	60:35:5:0.5	50:50:0:0.5	100:0:0:0	100:0:0:0	100:0:0:0	100:0:0:0	100:0:0: 0.1
	**Areal capacity (mAh cm^{-2})**	3.77	3.9	-	-	-	-	7
	**Areal mass (mg cm^{-2})**	17	21.1	4.3	92.8	5.3	73.1	2.0
	**N/P ratio**	1.3	1.3	-	-	-	-	1.4
**Cell**	**Al thickness (μm)**	10	10	10	10	10	10	10
	**Cu thickness (μm)**	10	10	10	10	10	10	10
	**Total areal mass (mg cm^{-2})**	70.3	66.5	37.9	118.4	46.6	98.2	53.2
	**Total thickness (μm)**	339.2	254.4	196.1	433.0	254.4	190.2	205
	**Chemistry**	Gr || NCM9055	Gr || LCO	Li || NCM523	LiIn || S	Li || NCM523	LiIn || NCM622	Si || NCM82
	**Nominal cell voltage (V)**	3.55	3.55	3.7	1.375	3.7	3.075	3.35
**Performance**	**Cell format**	Pouch	Pouch	Pellet/Coin	Pellet/Coin	Pellet/Coin	Pellet/Coin	Pouch
	**Current density (mA cm^{-2})**	0.7 (0.24 C)	0.3 (0.1 C)	0.085 (0.05 C)	0.4 (0.33 C)	0.192 (0.2 C)	0.64 (1 C)	0.5 (0.1 C)
	**Capacity utilization (%)**	62.5	88.8	84.6	95	86.3	72.8	82.6
	**Cycle number**	100	50	150	500	50	1000	30
	**Capacity retention (%)**	93.6	68.6	80.1	80.8	88.4	86.4	83.2
	**Specific energy (Wh kg^{-1})**	146.3	160.2	181.1	13.9	76.2	20.0	310
	**Energy density (Wh L^{-1})**	303.5	418.6	350.1	38.1	139.6	103.5	805

Supplementary Table 2 | EIS equivalent circuit fitting values of Li^+ transport in SSE layer of co-rolled film and freestanding films in Fig. 4c.

Co-rolled film				Freestanding films			
Element	Value	Error	Unit	Element	Value	Error	Unit
R1	6.098	0.2056	Ω	R1	49.48	0.5371	Ω
CPE1-T	3.69E-06	8.01E-08	F s^{P-1}	CPE1-T	6.51E-06	7.11E-08	F s^{P-1}
CPE1-P	0.80011	0.002965	-	CPE1-P	0.80558	0.001974	-
$\chi^2 = 0.01502$				$\chi^2 = 0.004286$			

Supplementary Table 3 | EIS equivalent circuit fitting values of Li^+ transport in positive electrode layer of co-rolled film and freestanding films in Fig. 4e.

Co-rolled film				Freestanding films			
Element	Value	Error	Unit	Element	Value	Error	Unit
R1	68.26	2.7599	Ω	R1	70.9	0.74489	Ω
R2	112.4	10.077	Ω	R2	111.7	6.6145	Ω
CPE2-T	0.00047836	1.2205E-05	F s^{P-1}	CPE2-T	0.00058069	1.1092E-05	F s^{P-1}
CPE2-P	0.80737	0.021546	-	CPE2-P	0.80899	0.014236	-
R3	72.84	14.889	Ω	R3	92.45	8.3963	Ω
CPE3-T	0.0021416	0.00022355	F s^{P-1}	CPE3-T	0.0021085	0.00013923	F s^{P-1}
CPE3-P	0.40015	0.022624	-	CPE3-P	0.39997	0.018399	-
CPE4-T	0.064197	0.021041	F s^{P-1}	CPE4-T	0.17251	0.1447	F s^{P-1}
CPE4-P	0.077914	0.031419	-	CPE4-P	0.17043	0.083097	-
χ^2 = 0.00032217				χ^2 = 0.00011788			

Supplementary Table 4 | Summary of binders used in dry-process and potential compatibility with co-rolling dry-process based on the criteria of binder property, binding type, and fabrication method.

**Binder**	Polytetrafluoro ethylene (PTFE)	Polyvinylidene fluoride (PVDF)	Paraffin	Ethylene-vinyl acetate (EVA)	Hydrogenated nitrile butadiene rubber (HNBR)	Styrene-butadiene rubber (SBR)
**Binder property**	Thermoplastic	Thermoplastic	Thermoplastic	Thermoplastic	Thermoset elastomer	Thermoset elastomer
**Binding type**	Fibrillated	Melted	N/A	Fibrillated	N/A	N/A
**Fabrication method**	Roll/Shear	Dry spray, Mold-press	Roll/Shear	Roll/Shear	Roll/Shear	Roll/Shear
**Compatibility with co-rolling**	Compatible (This work)	Potentially not compatible	Potentially less compatible	Potentially compatible	Potentially less compatible	Potentially less compatible
**Reference**	1, 2	3, 4	5	6	7	8

Supplementary Note 1 | Specific energy and energy density calculations of co-rolled film.

Specific energy (Wh kg^{-1}) and energy density (Wh L^{-1}) in Fig. 6f were calculated based on stack-level (including SSE, positive and negative electrodes, and Al and Cu current collectors). The obtained CAM-level specific energy was converted to stack-level specific energy or energy density by using the areal mass and thickness values of each layer stated in Supplementary Table 1. Specifically, areal masses of 8.2, 31.4, 2.0, 2.7, 8.96 mg cm^{-2} and thicknesses of 50, 120, 15, 10, 10 μm were used for SSE, positive electrode, negative electrode, Al, and Cu, respectively. This corresponds to a total areal mass of 53.2 mg cm^{-2} and thickness of 205 μm of a cell stack.

Supplementary References:

1. Zhang, Z., Wu, L., Zhou, D., Weng, W. & Yao, X. Flexible Sulfide Electrolyte Thin Membrane with Ultrahigh Ionic Conductivity for All-Solid-State Lithium Batteries. *Nano Lett.* **21**, 5233−5239 (2021).
2. Hippauf, F. *et al.* Overcoming binder limitations of sheet-type solid-state cathodes using a solvent-free dry-film approach. *Energy Storage Mater.* **21**, 390−398 (2019).
3. Ludwig, B., Zheng, Z., Shou, W., Wang, Y. & Pan, H. Solvent-Free Manufacturing of Electrodes for Lithium-ion Batteries. *Sci. Rep.* **6**, 23150 (2016).
4. Ryu, M., Hong, Y.-K., Lee, S.-Y. & Park, J. H. Ultrahigh loading dry-process for solvent-free lithium-ion battery electrode fabrication. *Nat. Commun.* **14**, 1316 (2023).
5. Kim, M. K. *et al.* Fluorine-Free Paraffin Binder-Based Dry Thick Electrodes Toward Sustainable and Efficient Battery Manufacturing. (2024) doi:10.2139/ssrn.4951163.
6. Zhu, X., Jiang, W., Wang, L. & Lu, J. Constructing Resilient Cross-Linked Network Toward Stable All-Solid-State Lithium-Sulfur Batteries. *Adv. Energy Mater.* **14**, (2024).
7. Khakani, S. E. *et al.* Melt-processed electrode for lithium ion battery. *J. Power Sources* **454**, 227884 (2020).
8. Li, Y. *et al.* Long-Life Sulfide All-Solid-State Battery Enabled by Substrate-Modulated Dry-Process Binder. *Adv. Energy Mater.* **12**, 2201732 (2022).
