# Supplementary material for: Robust interface and reduced operation pressure enabled by co-rolling dry-process for stable all-solid-state batteries
Source: Nat Commun. 2025 May 6;16:4200. doi: 10.1038/s41467-025-59363-4 (PMC12055973; doi:10.1038/s41467-025-59363-4)
Supplement: Supplementary file 3 — Description of Additional Supplementary Files [file 41467_2025_59363_MOESM3_ESM.pdf]

<u>Description of Additional Supplementary Files</u>

Supplementary Movie 1. Peel-off test of freestanding SSE and positive electrode films after lamination.

Supplementary Movie 2. Peel-off test of co-rolled SSE and positive electrode film.
